# A modular system of DNA enhancer elements mediates tissue-specific activation of transcription by high dietary zinc in *C. elegans*

**DOI:** 10.1093/nar/gku1360

**Published:** 2014-12-30

**Authors:** Hyun Cheol Roh, Ivan Dimitrov, Krupa Deshmukh, Guoyan Zhao, Kurt Warnhoff, Daniel Cabrera, Wendy Tsai, Kerry Kornfeld

**Affiliations:** 1 Department of Developmental Biology, Washington University School of Medicine, St. Louis, MO 63110, USA; 2 Department of Pathology and Immunology, Washington University School of Medicine, St. Louis, MO 63110, USA

## Abstract

Zinc is essential for biological systems, and aberrant zinc metabolism is implicated in a broad range of human diseases. To maintain homeostasis in response to fluctuating levels of dietary zinc, animals regulate gene expression; however, mechanisms that mediate the transcriptional response to fluctuating levels of zinc have not been fully defined. Here, we identified DNA enhancer elements that mediate intestine-specific transcriptional activation in response to high levels of dietary zinc in *C. elegans*. Using bioinformatics, we characterized an evolutionarily conserved enhancer element present in multiple zinc-inducible genes, the high zinc activation (HZA) element. The HZA was consistently adjacent to a GATA element that mediates expression in intestinal cells. Functional studies using transgenic animals demonstrated that this modular system of DNA enhancers mediates tissue-specific transcriptional activation in response to high levels of dietary zinc. We used this information to search the genome and successfully identified novel zinc-inducible genes. To characterize the mechanism of enhancer function, we demonstrated that the GATA transcription factor ELT-2 and the mediator subunit MDT-15 are necessary for zinc-responsive transcriptional activation. These findings define new mechanisms of zinc homeostasis and tissue-specific regulation of transcription.

## INTRODUCTION

Zinc is a metal that is essential in all biological systems. Zinc is involved in a wide range of cellular processes; Zn^2+^ is a structural and/or enzymatic cofactor in a large number of proteins (1), and Zn^2+^ is a signaling molecule during development, immune responses and synaptic transmission (2–4). Zinc is essential for human health, and abnormalities of zinc metabolism are implicated in several diseases. Zinc deficiency due to inadequate dietary intake or genetic disorders that disrupt zinc uptake causes a wide range of abnormalities in multiple tissues including the skin and immune system (5–7). Excess zinc is also deleterious to humans, and zinc may mediate cell death following ischemic injury (8,9). Furthermore, genetic variations that affect zinc metabolism have been correlated with a variety of human diseases, such as cancer, diabetes and neurodegenerative diseases (10–12). Understanding mechanisms that regulate zinc metabolism is important for biology and human health.

In eukaryotic cells, zinc metabolism is regulated by zinc transporters and zinc-binding molecules. Zinc transporters mediate the movement of zinc ions across membranes and belong to two major families, Cation Diffusion Facilitator (CDF, also known as ZnT or SLC30) and Zrt-, Irt-like protein (ZIP, also known as SLC39) (13). CDF proteins function to lower the concentration of cytoplasmic zinc by exporting zinc across the plasma membrane into the extracellular space or sequestering zinc into the lumen of intracellular organelles. By contrast, ZIP proteins function to increase the concentration of cytoplasmic zinc by importing zinc across the plasma membrane or releasing zinc from intracellular organelles. Mammals contain 10 CDF and 14 ZIP proteins, and each protein displays a unique tissue distribution and subcellular localization (14). It is thought that the cytoplasm has a very low concentration of free zinc ions, since most exchangeable zinc ions appear to be sequestered in intracellular organelles or bound to small molecules or small proteins, such as metallothionein. Metallothioneins belong to a family of small cytosolic proteins that can bind zinc and other physiological metal ions through cysteine residues, and metallothioneins have been proposed to function in zinc detoxification and storage (15).

To maintain zinc homeostasis in response to fluctuating zinc levels, organisms regulate the abundance and activity of zinc transporters and metallothioneins. For example, exposure to high levels of zinc causes repression of specific ZIP proteins, thereby reducing zinc uptake (16), and induction of specific CDF proteins and metallothioneins, thereby increasing zinc excretion and sequestration in intracellular organelles and proteins; these homeostatic adjustments protect against zinc toxicity (17). The coordinated response of multiple proteins is regulated at the transcription level, and zinc-responsive transcription factors have been characterized in several organisms. In the yeast, *Saccharomyces cerevisiae*, the zinc-responsive activator protein1 (ZAP1), a transcription factor that contains multiple zinc finger domains, is repressed by high zinc (18) and activated by low zinc; ZAP1 induces target gene expression by directly binding a conserved DNA element in promoter regions, 5′-ACCYYNAAGGT-3′, called the zinc-responsive element (ZRE) (19). The zinc importers ZRT1 and ZRT2 are induced by ZAP1 to promote zinc uptake, and ZRT3 is induced to mobilize zinc stored in the vacuole (20). In the fission yeast, *Schizosaccharomyces pombe*, the Loz1 transcription factor was recently discovered to be required for gene repression in zinc-replete cells (21,22). In mammals, the metal-responsive-element-binding transcription factor-1 (MTF-1), a zinc-finger containing transcription factor, is activated by high zinc (23). Activated MTF-1 translocates into the nucleus and induces expression of target genes that contain the conserved DNA element, 5′-TGCRCNCGGCCC-3′, called the metal response element (MRE). MTF-1 induces metallothioneins, MT-I and MT-II, and the zinc exporter ZnT1 to promote zinc sequestration and excretion, respectively (24,25). MTF-1 can also negatively regulate transcription; for example, MTF-1 represses ZIP10 transcription by binding an MRE located downstream of the transcription start site (26). A distinct zinc-responsive *cis*-regulatory element was recently reported in humans, called the zinc transcriptional regulatory element; this element functions independently of MTF-1, and transcription factors that bind this element have yet to be identified (27). A deletion of yeast ZAP1 causes defective zinc homeostasis (18), and a deficiency of murine MTF-1 causes embryonic lethality (28), indicating these transcription factors have critical functions in zinc metabolism and development.

The nematode *Caenorhabditis elegans* is a useful and relevant model organism to study zinc metabolism in animals. Powerful genetic techniques and a simple body plan have facilitated the elucidation of mechanisms of zinc metabolism (29–34). The *C. elegans* genome encodes evolutionarily conserved zinc transporters and metallothioneins, suggesting studies of *C. elegans* are relevant to mammalian biology (13,35). Furthermore, the expression of *C. elegans* zinc transporters and metallothioneins is regulated in response to dietary zinc levels, similar to yeast and mammals. The CDF genes *cdf-2* and *ttm-1b* are transcriptionally upregulated by high dietary zinc in intestinal cells, which promotes zinc storage and excretion (17,31). *C. elegans* contains two metallothionein genes, *mtl-1* and *mtl-2*, that are induced at the level transcription by high dietary zinc in intestinal cells (36). The GATA-type transcription factor ELT-2 has been implicated in metallothionein expression in *C. elegans* (37). ELT-2 function is essential for the development of intestinal cells and promotes intestinal cell-specific gene expression (38). However, ELT-2 does not appear to be regulated by dietary zinc. These findings demonstrate that *C. elegans* regulates transcription to maintain zinc homeostasis, suggesting these animals have mechanisms to sense the level of dietary zinc and execute a transcriptional response. However, the mechanism of this response has not been well defined. The *C. elegans* genome encodes many zinc finger transcription factors, but none are clearly homologous to yeast ZAP1 or mammalian MTF-1, so candidate transcription factors have not been identified.

To characterize regulatory mechanisms that mediate zinc homeostasis, we identified DNA enhancer elements that mediate transcriptional activation in response to high levels of dietary zinc in *C. elegans*. By using bioinformatic approaches, we identified an evolutionarily conserved DNA element that is present in the promoter regions of multiple genes that are induced by high dietary zinc, which we named the *C. elegans* high zinc activation (HZA) element. The HZA element was necessary for transcriptional activation of multiple genes, and it was sufficient to mediate the activation of a heterologous promoter in response to dietary zinc. The HZA element was consistently adjacent to a GATA element, and the GATA element was also necessary for the response to high zinc. Using our definition of this *cis*-acting DNA element, we successfully predicted novel zinc responsive genes. To identify *trans* acting factors, we analyzed candidate genes and demonstrated that the ELT-2 transcription factor and the mediator subunit MDT-15 are necessary for zinc-mediated transcriptional induction. We propose that a modular system of DNA enhancer elements promotes the intestinal-specific induction of zinc responsive genes: the GATA element specifies intestinal expression and the HZA element specifies regulation by zinc. These findings elucidate new mechanism of zinc homeostasis and tissue-specific regulation of transcription in response to environmental cues.

## MATERIALS AND METHODS

### General methods and strains

*C. elegans* strains were cultured at 20°C on nematode growth medium (NGM) seeded with *Escherichia coli* OP50 (39) except as noted. For zinc supplementation, noble agar minimum medium (NAMM) dishes were supplemented with zinc sulfate (ZnSO_4_) and seeded with concentrated OP50 (30). The wild-type *C. elegans* and parent of all transgenic strains was Bristol N2.

### Identification of the HZA element and alignments with other nematode species

To identify DNA motifs in zinc-responsive genes from *C. elegans*, we used the computational tool Multiple Em for Motif Elicitation (MEME) with the default settings (40). The following sequences were analyzed: 520 bp upstream of the *mtl-1* coding sequence, 580 bp upstream of the *mtl-2* coding sequence, 1371 bp upstream of the *cdf-2* coding sequence and 1655 bp extending from −4067 to −2413 bp upstream of the *ttm-1b* coding sequence. To search for DNA motifs in cadmium-responsive genes from *C. elegans*, we selected 29 genes from the previously described list of genes induced more than 4-fold based on a microarray study following exposure to cadmium for 24 h (41). The promoter sequences used as inputs for the analysis were intergenic regions extending from the coding sequence upstream to the adjacent gene. The element with the lowest *E*-value was considered to be most significant.

To compare HZA elements between nematode species, we used data from the UCSC genome browser to identify homologous genes in *C. briggsae*,*C. remanei* and *C. brenneri.* We analyzed genomic DNA sequences positioned upstream of the coding sequence, including 1 kb fragments from each species, except 500 bp of *C. elegans mtl-1* and 600 bp of *C. elegans mtl-2*. Multiple and/or pairwise sequence alignments were conducted by Clustal X (42) to identify evolutionarily conserved sequences between species.

### Plasmid DNA construction and transgenic strain generation

To generate transcriptional fusion constructs of high zinc-activated genes, we polymerase chain reaction (PCR)-amplified DNA fragments positioned upstream of the coding sequences using wild-type genomic DNA and ligated these fragments into pBluescript SK+ (Stratagene) together with the green fluorescent protein (GFP) coding sequence and the *unc-54* 3′ untranslated region (UTR). To generate the *mtl-1* translational fusion constructs, we PCR-amplified the region extending from ∼800 bp upstream of the *mtl-1* start codon to the last exon of *mtl-1* (without the stop codon) and ligated the fragment into pBluescript SK+ in frame with the GFP coding sequence. Next, ∼3 kb of the *mtl-1* 3′UTR was PCR-amplified and inserted downstream of the GFP coding sequence. To generate mutated promoter sequences, we replaced the sequence of the HZA element (15 bp) or the GATA element (8 bp) of a wild-type promoter with a scrambled sequence of the same length using the method of fusion PCR. To generate *pes-10* promoter plasmids, we PCR-amplified the 62 bp sequence of *mtl-1* that contains the HZA and GATA elements and ligated this fragment into pPD107.94, a plasmid containing the *pes-10* minimal promoter. To insert three copies of this 62 bp sequence, we repeated this procedure twice using different restriction enzyme sites.

Transgenic animals containing extrachromosomal arrays were generated by injecting a plasmid mixed with the coinjection marker pCJF104 [*Pmyo-3::mCherry*] into wild-type animals (43). Transgenic animals were selected by mCherry expression in the body wall muscles. The plasmids and transgenic strains used in this study are described in Supplementary Table S1. For fluorescence microscopy, animals were paralyzed in a drop of 10 mM levamisole in M9 on a 2% agarose pad on a microscope slide, and fluorescence was visualized using a Zeiss Axioplan 2 microscope equipped with a Zeiss AxioCam MRm digital camera. To compare fluorescence between different zinc culture conditions, we captured images using the identical settings and exposure times.

### Quantitative real-time PCR (qRT-PCR)

We conducted qRT-PCR as previously described with minor modifications (31). Briefly, to obtain synchronized populations of animals, we isolated eggs from gravid adult hermaphrodites by bleaching, hatched the eggs in M9 overnight and cultured starved L1 larvae on NGM dishes for ∼3 days. Synchronized animals at adult stages were collected by washing and then cultured on NAMM dishes supplemented with zinc sulfate (ZnSO_4_) and seeded with concentrated OP50. After 16–24 h, animals were collected by washing for RNA isolation. RNA was isolated using TRIzol (Invitrogen) and treated with DNase I, and cDNA was synthesized using the High-Capacity cDNA Reverse Transcription kit according to the manufacturer's protocol (Applied Biosystems). PCR was performed using an Applied Biosystems 7900 thermocycler and SYBR Green PCR Master Mix (Applied Biosystems). Fold change was determined by comparing the changes of target gene expression between different conditions with the changes of the reference gene expression (*rps-23* for endogenous genes or *mCherry* for *GFP* transgenes) under the same conditions.

### Identification of new genes containing HZA and GATA elements

The chromosomal sequence and the gene structures of *C. elegans* (CONSORTIUM. 1998)(WS228) were downloaded from the WormBase ftp-site (ftp://ftp.wormbase.org/pub/wormbase/releases/). Using the Patser software (44), we identified all predicted sites in the upstream regions for the HZA element (generated by analyzing *mtl-1*,*mtl-2*,*cdf-2* and *ttm-1b*) and GATA element (generated by analyzing intestine-enriched genes) (38) using default cutoff scores. We analyzed upstream intergenic region sequences of up to 20 kb in length; if the distance to the upstream gene is less than 20 kb, then only the intergenic region was obtained. Next, we searched for genes that meet the following three criteria: (i) contain both a HZA element and a GATA element, (ii) the HZA element and GATA element are both within the c*is*-regulatory modules predicted by *C. elegans* Regulatory Module Detector (45), (iii) the distance between the predicted HZA element and GATA element is less than or equal to 20 bp. This analysis identified 24 genes (Figure 6B). In addition, we performed the analysis using only criteria 1 and 3 and identified 116 genes (Supplementary Table S2).

**Figure 1.**
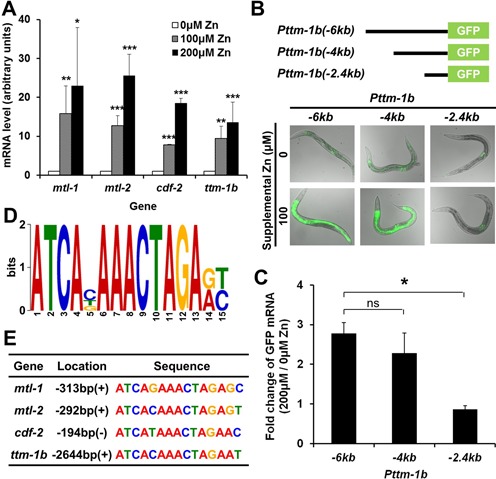
Identification of *C. elegans* HZA element. (A) Wild-type animals were cultured with 0, 100 or 200 μM supplemental zinc. RNA was extracted from a synchronized population at L4/adult stages, and mRNA levels of indicated genes were determined by qRT-PCR. The bars indicate mRNA levels at 0, 100 and 200μM supplemental zinc; mRNA levels at 0 μM supplemental zinc were set equal to 1.0 for each gene. Values are the average ± SEM of four independent experiments. All four genes displayed statistically significant increases at 100 and 200 μM supplemental zinc (*P < 0.05, **P < 0.01, ***P < 0.005). (B) Diagrams (not to scale) show the extent of DNA from the *ttm-1b* gene (black line) upstream of the translation start site fused to the coding region of GFP (green box). Images show transgenic animals containing these plasmids that were cultured with 0 or 100 μM supplemental zinc at L4/adult stage and imaged by fluorescence microscopy. GFP fluorescence signals (green) were captured with the identical settings and exposure times and overlaid with bright field images. (C) Transgenic animals containing reporter constructs shown in panel B were cultured with 0 or 200 μM supplemental zinc, and GFP mRNA levels were analyzed by qRT-PCR. The bars indicate the fold change of GFP mRNA levels as the ratio between the level at 200 and 0 μM supplemental zinc. A positive value indicates induction in high zinc. Values are the average ± SEM of three independent experiments. The −2.4 kb promoter displayed significantly reduced induction compared to the −6-kb promoter (*P < 0.05). (D) A sequence logo (position weight matrix) of the HZA element identified by MEME using the promoter regions of *C. elegans mtl-1, mtl-2, cdf-2* and *ttm-1b*. The height of the nucleotide at each position represents the frequency in the four DNA sequences scaled in bits. (E) The sequence of the HZA element in *mtl-1, mtl-2, cdf-2* and *ttm-1b*. The location is the first nucleotide of the DNA motif relative to the translation start site (ATG); (+) and (−) indicate the same and opposite orientation relative to the direction of transcription, respectively.

**Figure 2.**
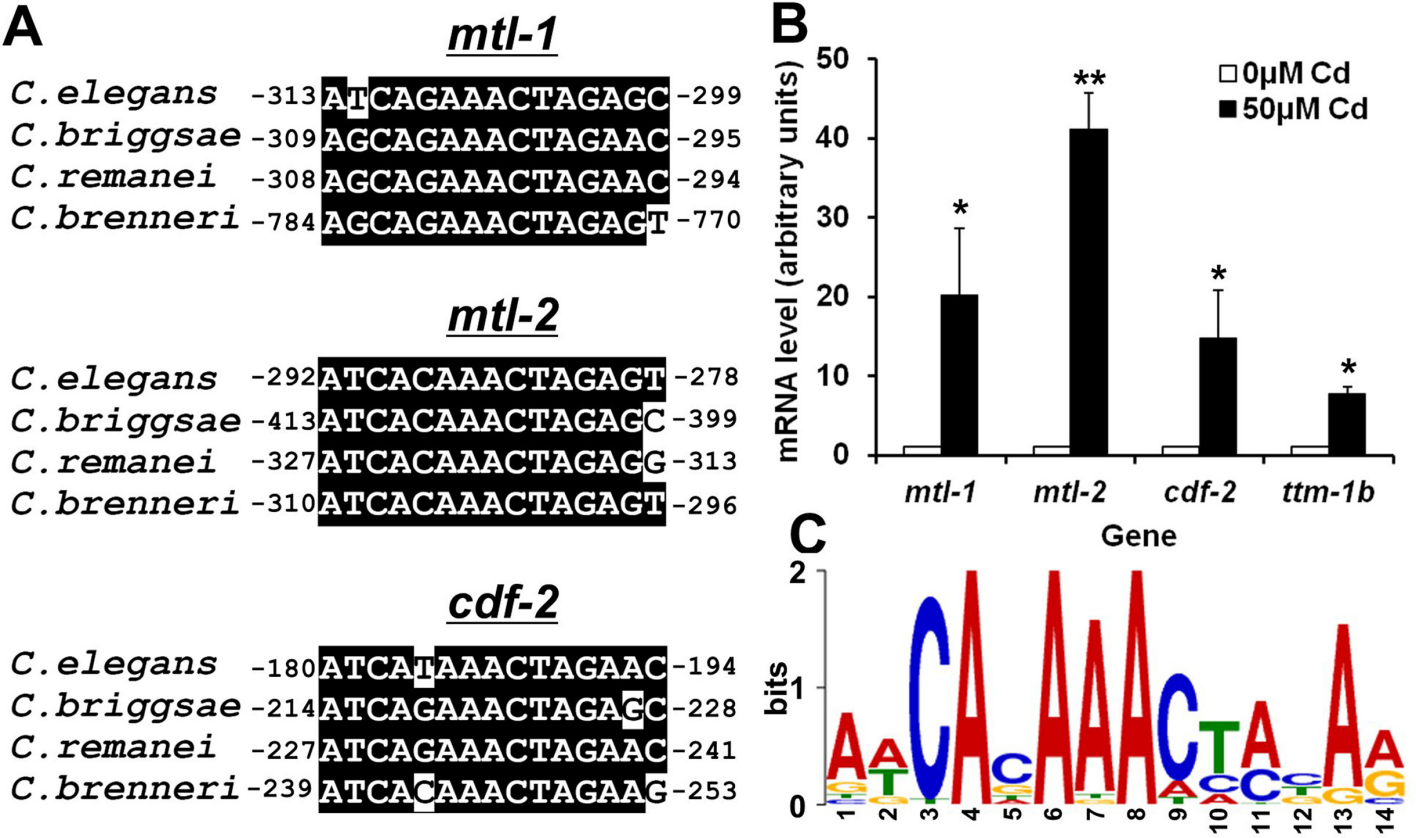
The HZA element is conserved in nematodes and present in cadmium-responsive genes. (A) Sequence alignments of the HZA elements in the promoter regions of *mtl-1, mtl-2* and *cdf-2* from the *Caenorhabditis* species *elegans, briggsae, remanei* and *brenneri*, analyzed by Clustal X. Identical nucleotides are highlighted in black, and numbers indicate the position of the first nucleotide relative to the start of translation. (B) The bars indicate mRNA levels from wild-type animals cultured with 0 and 50 μM supplemental cadmium that were analyzed by qRT-PCR; mRNA levels at 0 μM supplemental cadmium were set equal to 1.0 for each gene. Values are the average ± SEM of two independent experiments. All four genes displayed statistically significant increases at 50 μM supplemental cadmium (*P < 0.05, **P < 0.01). (C) A sequence logo (position weight matrix) of the DNA element identified by MEME using the promoter regions of 29 cadmium inducible genes from *C. elegans* (Supplementary Figure S1). The height of the nucleotide at each position represents the frequency of that nucleotide in the 29 sequences.

**Figure 3.**
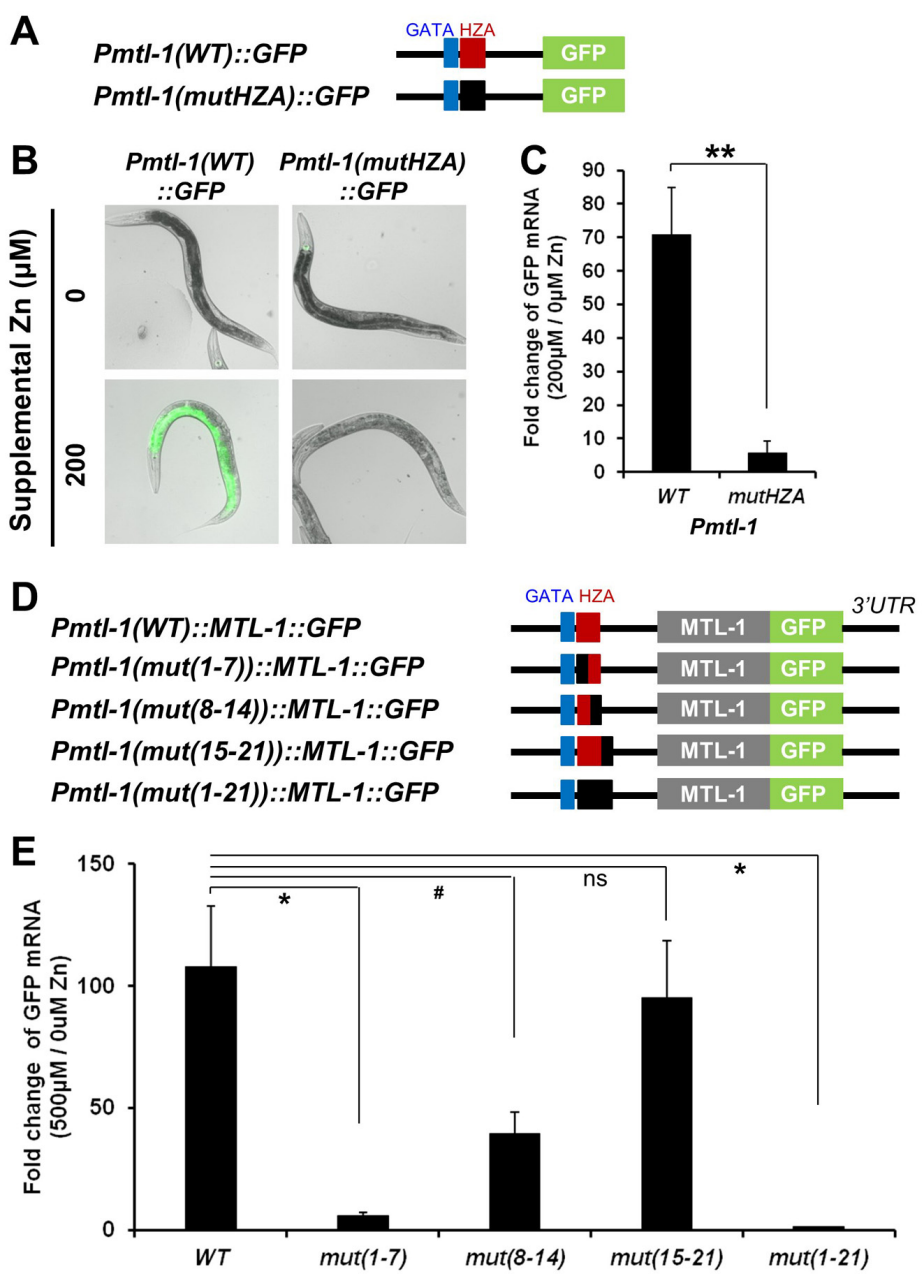
The HZA element is necessary for *mtl-1* induction in response to high dietary zinc. (A and D) Diagrams (not to scale) of the *mtl-1* promoter region extending 489 bp upstream of the translation start codon (black line) that was fused to the coding region for GFP (panel A) or had the coding region of GFP inserted at the position of the stop codon (panel D). The fragment includes the wild-type GATA element (blue box), and the wild-type HZA element (red box) or mutated HZA elements (black box) consisting of scrambled nucleotide sequence. The MTL-1 coding region (gray box) and the 3′UTR are shown in panel D. (B) Images show transgenic animals containing the indicated plasmids that were cultured with 0 or 200 μM supplemental zinc at L4/adult stage and imaged by fluorescence microscopy. GFP fluorescence signals (green) were captured with the identical settings and exposure times and overlaid with bright field images. (C) Transgenic animals containing reporter constructs shown in panel A were cultured with 0 or 200 μM supplemental zinc, and GFP mRNA levels were analyzed by qRT-PCR. The bars indicate the fold change of GFP mRNA levels as the ratio between the level at 200 and 0 μM supplemental zinc. A positive value indicates induction in high zinc. Values are the average ± SEM of three independent experiments. The mutated promoter was significantly different than the wild-type promoter (**P < 0.01). (D and E) Transgenic animals containing reporter constructs shown in panel D were cultured with 0 or 500 μM supplemental zinc, and GFP mRNA levels were analyzed by qRT-PCR. The bars indicate the fold change of GFP mRNA levels as the ratio between the level at 500 and 0 μM supplemental zinc. A positive value indicates induction in high zinc. Values are the average ± SEM of three independent experiments. The mutated promoters are compared to the wild-type promoter (*P < 0.05, #*P* = 0.06, ns *P* = 0.73). 500 μM supplemental zinc is a high concentration that causes toxicity with continuous culture; a short exposure to this high concentration of zinc was used in this experiment to maximize the response of mutant promoters and improve our ability to measure a small level of induction.

**Figure 4.**
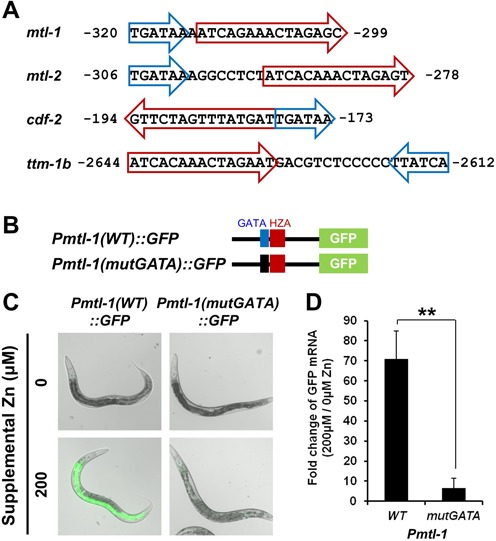
GATA element was necessary for regulation of *mtl-1* by dietary zinc. (A) Fragments of the promoters of *mtl-1, mtl-2, cdf-2* and *ttm-1b*. Red arrows indicate the HZA element, and blue arrows indicate the GATA element; the direction of the arrows indicates the orientation of the elements, and numbers show the distance from the translation start site. (B) Diagrams (not to scale) of the *mtl-1* promoter region extending 489 bp upstream of the translation start codon (black line) that was fused to the coding region for GFP. The fragment includes the wild-type HZA element (red box) and the wild-type GATA element (blue box) or the mutated GATA element (black box) consisting of scrambled nucleotide sequence. (C) Fluorescence microscope images of transgenic animals at L4/adult stages containing the indicated promoter constructs and cultured with 0 or 200 μM supplemental zinc. Bright field images are overlaid with GFP fluorescence signal (green) that were captured with the identical settings and exposure times. (D) Transgenic animals containing reporter constructs shown in panel B were cultured with 0 or 200 μM supplemental zinc, and GFP mRNA levels were analyzed by qRT-PCR. The bars indicate the fold change of GFP mRNA levels as the ratio between the level at 200 and 0 μM supplemental zinc. A positive value indicates induction in high zinc. Values are the average ± SEM of three independent experiments. The mutated promoter displayed significantly reduced induction compared to wild type (**P < 0.01).

**Figure 5.**
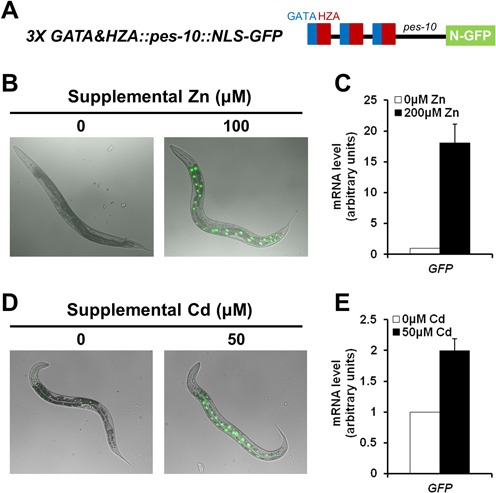
A combination of the HZA and GATA elements was sufficient to induce intestine-specific transcriptional activation in response to high zinc. (A) A diagram (not to scale) of the *pes-10* minimal promoter containing three tandem copies of a 62-bp fragment of the *mtl-1* promoter that includes a wild-type GATA element (blue box) and the wild-type HZA element (red box) driving expression of GFP containing a nuclear localization signal (N-GFP). (B and D) Fluorescence microscope images of transgenic animals at L4/adult stages containing the *pes-10* promoter shown above and cultured with 0 or 100 μM supplemental zinc (B) or 0 or 50 μM supplemental cadmium (D). Bright field images are overlaid with GFP fluorescence signal (green) that were captured with the identical settings and exposure times. (C and E) Transgenic animals were cultured with 0 or 200 μM supplemental zinc (C) or 0 or 50 μM supplemental cadmium (E). RNA was extracted from a synchronized population at L4/adult stages, and bars indicate GFP mRNA levels determined by qRT-PCR. mRNA levels at 0 μM supplemental zinc or cadmium were set equal to 1.0. Values are the average ± SEM of three independent experiments. GFP mRNA was consistently elevated at 200 μM supplemental zinc or 50 μM supplemental cadmium in three independent trials, indicating this is a reproducible result. However, the combined data did not reach statistical significance at the level of *P* < 0.05 because the values of the fold changes varied between the experiments.

**Figure 6.**
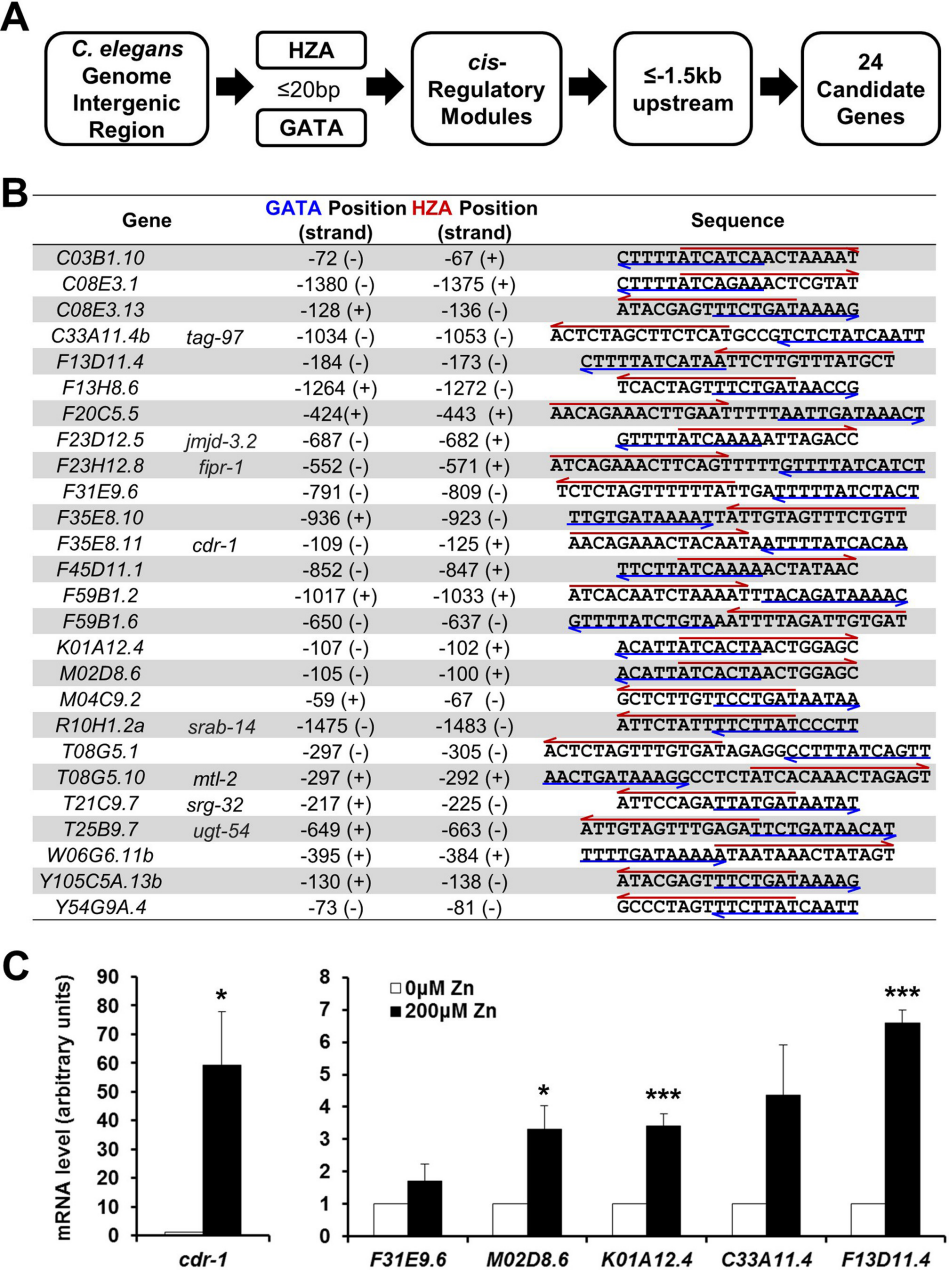
Bioinformatic identification of new genes activated by high zinc. (A) A diagram of the steps used to identify candidate genes. (i) Identify all intergenic regions in the *C. elegans* genome. (ii) Identify the subset that contains both a HZA and a GATA element separated by less than 20 bp. To search for the GATA element, we used a 12-bp weight matrix, and to search for the HZA element, we used a 15-bp weight matrix. (iii) Identify the subset that have the HZA and GATA element located in a conserved *cis*-regulatory module (45). (iv) Identify the subset where the module is located <1.5 kb upstream of the translation start codon. (B) Twenty-four candidate genes met these criteria. Each gene has a genomic designation, and eight genes also have a genetic name (right). Numbers indicate the position of the upstream base pair of the GATA element or the HZA element. Positions are relative to the predicted translation start site of the gene (ATG), where the A is defined as +1 and the preceding nucleotide is defined as −1. (+) and (−) indicate the same and opposite orientation relative to the direction of transcription, respectively. The HZA and GATA elements are indicated by a red arrow over and blue arrow under the nucleotide sequence, respectively. (C) Wild-type animals were cultured with 0 or 200 μM supplemental zinc. RNA was extracted from a synchronized population at L4/adult stages, and mRNA levels of indicated genes were determined by qRT-PCR. The bars indicate mRNA levels at 0 and 200 μM supplemental zinc; mRNA levels at 0 μM supplemental zinc were set equal to 1.0 for each gene. Values are the average ± SEM of three independent experiments. The scales differ in the left and right panels. Comparisons are to the same gene at 0 μM supplemental zinc (*P < 0.05, **P < 0.01, ***P < 0.005).

### RNA interference (RNAi)

RNAi experiments were performed as previously described (33). Briefly, RNAi bacteria were cultured in lysogeny broth (LB) containing carbenicillin overnight. The bacterial culture was diluted into LB containing carbenicillin and cultured for ∼6 h, and the bacteria were seeded on NGM dishes containing carbenicillin and isopropyl β-D-1-thiogalactopyranoside (IPTG) (1 mM) and dried overnight. Feeding wild-type hermaphrodites *elt-2* or *mdt-15* RNAi caused embryonic lethality of their progeny. To avoid causing lethality, we cultured animals at the L1 stage on NGM dishes seeded with RNAi bacteria. The control bacterial strain (HT115) contained the empty L4440 vector.

### Statistical analysis

All data were analyzed using the two-tailed unpaired Student's *t-*test, and differences with *P* < 0.05 were considered to be significant.

## RESULTS

### Identification of a conserved DNA sequence in the promoters of genes induced by high zinc

To quantify changes of mRNA levels in response to high dietary zinc, we synchronized wild-type animals at the fourth larval (L4) or adult stage, cultured these animals on NAMM containing 0, 100 or 200 μM supplemental zinc (30) for 14–16 h and analyzed mRNA levels of *mtl-1*,*mtl-2*,*cdf-2* and *ttm-1b* using qRT-PCR. All four genes displayed significant and dose-dependent increases in mRNA levels; levels increased 10- to 30-fold when cultured with 200 μM supplemental zinc compared to 0 μM supplemental zinc (Figure 1A). Thus, high levels of dietary zinc caused increased levels of mRNA accumulation for these four genes, suggesting that high levels of dietary zinc increased the synthesis and/or decreased the degradation of these mRNAs.

To determine how promoter sequences upstream of the translation start site contribute to this regulation, we dissected the *ttm-1* gene. *ttm-1* generates two transcripts that utilize distinct start sites: the *ttm-1a* transcript that is not zinc inducible and the *ttm-1b* transcript that is zinc inducible. Although *ttm-1b* is expressed in multiple cell types, the induction in response to zinc only occurs in intestinal cells (17). To focus on the *ttm-1b* promoter region, we analyzed transgenic animals containing a plasmid with ∼6 kb of DNA upstream of the *ttm-1b* translation start site driving expression of GFP, but lacking the *ttm-1b* coding region. Consistent with our previous report (17), this ∼6 kb region was sufficient to promote increased GFP accumulation in response to high dietary zinc (Figure 1B). To define fragments of this ∼6 kb region that are necessary for the response to dietary zinc, we generated plasmids with ∼4 and ∼2.4 kb of promoter DNA, and introduced these plasmids into transgenic animals. Transgenic animals containing the ∼4 kb promoter fragment displayed GFP that was widely expressed and specifically induced in intestinal cells by 100 μM supplemental zinc, similar to expression mediated by the ∼6-kb promoter fragment (Figure 1B). Quantitative analysis of GFP mRNA levels using qRT-PCR demonstrated that the induction of GFP is also observed at the mRNA level and was similar with ∼6 and ∼4 kb reporter constructs (Figure 1C). By contrast, transgenic animals containing the ∼2.4-kb promoter fragment displayed no GFP expression in intestinal cells, but did display GFP expression in other tissues (Figure 1B). The addition of 100 μM supplemental zinc did not alter GFP expression at the protein or mRNA level (Figure 1B and C). These results indicate that the region from −4 to −2.4 kb upstream of *ttm-1b* translation start site contains *cis*-regulatory elements that are necessary for intestine-specific transcriptional induction in response to high levels of dietary zinc, whereas the sequences necessary for non-intestinal expression appear to be contained in the ∼2.4-kb fragment. Furthermore, zinc induction can be conferred on the GFP transcript, indicating that regulation is likely to occur at the level of mRNA synthesis rather than degradation.

We reasoned that a similar DNA sequence might regulate the zinc inducibility of all four genes. To identify such a sequence, we analyzed the regions upstream of the translation start sites of *mtl-1, mtl-2* and *cdf-2* and the *ttm-1b* promoter region between −4 and −2.4 kb. Using the computational tool MEME (40), we identified DNA motifs present in all four zinc-responsive genes. Figure 1D shows the highest scoring motif identified by MEME, and we named this element the HZA element. The HZA element was located 313, 292, 194 and 2644 bp upstream of the start codons of *mtl-1, mtl-2, cdf-2* and *ttm-1b*, respectively (Figure 1E). In *mtl-1*,*mtl-2* and *ttm-1b*, the HZA element was located on the (+) strand, while it was located on the (−) strand of *cdf-2* (Figure 1E). Based on these results, we hypothesized that the HZA element functions in multiple genes to promote induction of mRNA levels in intestinal cells in response to high dietary zinc.

### The HZA element is conserved in nematodes and present in cadmium-inducible genes

To begin to evaluate the functional relevance of the HZA element, we determined if it is evolutionarily conserved in three other nematode species that have available genome sequences: *C. briggsae*,*C. brenneri* and *C. remanei*. We performed sequence alignment analysis with the presumptive promoter DNA sequences of four zinc-responsive genes from all four nematode species. We detected a HZA element in the promoter regions of *mtl-1*,*mtl-2* and *cdf-2* in all of the nematode species, and the DNA sequence of the HZA element was highly conserved (Figure 2A). We did not detect a HZA element in *ttm-1b* from the other nematode species; however, the *ttm-1* gene has a complex structure with two promoters in *C. elegans*, and that may affect our ability to find homologous elements (17). The identification of evolutionarily conserved HZA elements in three genes indicates that the element has functional significance.

To begin to characterize the specificity of the HZA element, we analyzed expression of the four zinc-inducible genes in response to other metals. We selected cadmium (Cd) and copper (Cu), since cadmium is a non-physiological metal that is physically and chemically similar to zinc, whereas copper is a physiological metal. Wild-type animals were cultured in the presence of 50 μM cadmium or 200 μM copper, and mRNA levels of *mtl-1*,*mtl-2*,*cdf-2* and *ttm-1b* were analyzed using qRT-PCR. Copper did not significantly affect gene expression (data not shown), indicating that there is some specificity in this transcriptional response. By contrast, cadmium significantly induced the expression of all the genes (Figure 2B). These results suggest that the HZA element may mediate responses to zinc and cadmium, which are similar, but not mediate a response to all transition metals.

To explore the role of the HZA element in the transcriptional response to cadmium, we analyzed a list of *C. elegans* cadmium-inducible genes defined by Cui *et al.* (41). The gene list included *mtl-1*,*mtl-2* and *cdf-2*, which displayed ∼15-, 32- and 4-fold increased expression after 24 h exposure to cadmium, respectively (41). We selected 29 of the cadmium-inducible genes that displayed similar levels of induction, ≥4-fold increased expression and searched for conserved DNA motifs using MEME. MEME identified a 14-bp DNA element that had the most statistically significant score, and a version of this DNA element was detectable in all 29 cadmium-inducible genes (Supplementary Figure S1). Figure 2C shows the sequence logo of this DNA element. This element completely overlapped with the HZA element in *mtl-1*,*mtl-2* and *cdf-2*, with the exception that position 15 no longer displayed significance. (Figures 1D and 2C). These results suggest that this DNA element may play a functional role in the induction of gene expression in response to cadmium. The sequence logo derived from aligning these 29 cadmium-responsive genes demonstrates the highest frequency of CA at position 3–4 and AAA at position 6–8, suggesting that these nucleotides may play the most important functional roles (Figure 2C).

### The HZA element was necessary for transcriptional activation in response to high zinc

To determine if the HZA element is required for transcriptional regulation in response to high dietary zinc, we used site directed mutagenesis to disrupt the element. A plasmid containing 489 bp of the wild-type *mtl-1* promoter driving expression of GFP protein was generated and introduced into transgenic worms (Figure 3A). In the absence of supplemental zinc, transgenic animals displayed low level, variable GFP expression in the pharynx and no detectable GFP expression in intestinal cells (Figure 3B and data not shown). By contrast, in the presence of 200 μM supplemental zinc, worms displayed strong GFP expression in intestinal cells, whereas pharyngeal expression levels were not altered (Figure 3B). Quantitative analysis using qRT-PCR showed that GFP mRNA levels were increased ∼70-fold in response to 200 μM supplemental zinc compared to 0 μM supplemental zinc (Figure 3C). To analyze the function of the HZA element, we used site-directed mutagenesis to replace the 15-bp HZA with a scrambled nucleotide sequence, and generated transgenic worms with this plasmid (Figure 3A). In response to 200 μM supplement zinc, transgenic animals containing the mutant promoter displayed no detectable GFP expression (Figure 3B). qRT-PCR analysis demonstrated that these animals did express a low level of GFP mRNA, but the level of induction in response to supplemental zinc was strongly reduced (Figure 3C). These results indicate that the 489-bp promoter region of *mtl-1* is sufficient to mediate transcriptional induction in intestinal cells in response to dietary zinc, and the HZA element is necessary for this response.

To analyze the function of the HZA element in additional zinc-responsive genes, we characterized *cdf-*2 and *ttm-1b.* The HZA element is located 194 bp upstream of the translation start site of *cdf-2* (Figure 1E). We generated a plasmid that contains 204 bp of the *cdf-2* promoter driving expression of GFP (Supplementary Figure S2A). Transgenic animals containing this plasmid displayed very faint GFP expression in intestinal cells when cultured with no supplemental zinc, whereas GFP expression was readily detectable in intestinal cells when animals were cultured with 200 μM supplemental zinc; analysis of mRNA levels demonstrated ∼5-fold induction (Supplementary Figure S2B and C). Site-directed mutagenesis was used to replace the *cdf-2* HZA element with a scrambled nucleotide sequence. In transgenic animals containing this mutant promoter, the induction of GFP expression by supplemental zinc was not detectable (Supplementary Figure S2B and C). Thus, a 204-bp fragment of the *cdf-2* promoter that contains the HZA element was sufficient to mediate zinc-induced transcription in intestinal cells, and the HZA element was necessary for this response. To analyze *ttm-1b*, we used the plasmid with 4067 bp upstream of the translation start site driving expression of GFP, since this region was sufficient to mediate transcriptional induction in response to zinc, and generated a mutant version with the HZA element replaced by scrambled nucleotides (Supplementary Figure S2D). Mutating the HZA element in the *ttm-1b* promoter eliminated the increase in both GFP protein and GFP mRNA levels in intestinal cells (Supplementary Figure S2E and F). These results demonstrate that the HZA element is necessary for transcriptional induction in response to high zinc in three different genes, suggesting this is a general property of the HZA element.

To dissect the function of the HZA element in the context of an intact gene, we generated a plasmid that contains the *mtl-1* genomic locus including the promoter, exons, introns and 3′UTR. Site-directed mutagenesis was used to insert the GFP coding sequence, so that the locus expresses a MTL-1::GFP fusion protein (Figure 3D). qRT-PCR analysis demonstrated that MTL-1::GFP mRNA expression levels were increased ∼100-fold in response to 500 μM supplemental zinc (Figure 3E), indicating that the genomic locus is sufficient to mediate robust transcriptional activation of *mtl-1* in response to zinc. To dissect the function of the HZA element, we mutated nucleotides 1–7 or 8–14 of the HZA element by substituting a scrambled sequence. As a negative control, we mutated nucleotides 15–21, which are adjacent to the HZA element, and as a positive control we mutated nucleotides 1–21. Transgenic animals containing these promoters were analyzed by qRT-PCR. The induction of MTL-1::GFP mRNA expression levels in response to high dietary zinc was almost completely absent in transgenic animals with the 1–21 mutation, whereas animals with the 15–21 mutation displayed a robust induction similar to wild type (Figure 3E). mRNA induction was strongly and significantly reduced by the 1–7 mutation, whereas there was a trend toward a moderate reduction caused by the 8–14 mutation. These results suggest that highly conserved nucleotides 1–7 of the HZA element are necessary for zinc induction, whereas nucleotides 8–14 make a measurable but less important contribution.

### A GATA element is adjacent to the HZA element and necessary for transcriptional activation by high zinc

By examining DNA sequences adjacent to the HZA element, we discovered that the *mtl-1*,*mtl-2*,*cdf-2* and *ttm-1b* promoters all contained the sequence element TGATAA (Figure 4A). We refer to this sequence as the GATA element. The GATA element was positioned in close proximity to the HZA element, separated by 0–12 base pairs in these four genes. Compared to the HZA element, both orientations were observed and the element could be either upstream or downstream relative to the translation start site (Figure 4A). The core of this sequence element, GATA, is the binding site for GATA transcription factors (46), including *C. elegans* ELT-2, which is known to be a master regulator of intestinal cell identity (38). To determine the function of the GATA element in zinc-responsive transcriptional regulation, we used site-directed mutagenesis to replace 8 base pairs containing the GATA element with a scrambled sequence of base pairs. Transgenic animals containing the mutated GATA element in the *mtl-1* promoter displayed strongly reduced induction of GFP protein and mRNA in response to 200 μM supplemental zinc compared to the induction mediated by the wild-type promoter (Figure 4B–D). This result is consistent with a previous report that the GATA element is specifically necessary for *mtl-1* expression (37).

To investigate the function of the GATA element in additional zinc-responsive genes, we mutated this element in the promoters of *cdf-2* and *ttm-1b*. Transgenic animals containing the mutated promoters displayed reduced induction of both GFP protein and mRNA in response to 200 μM supplemental zinc compared to transgenic animals containing wild-type promoters (Supplementary Figure S3). Importantly, the mutation in the *ttm-1b* promoter did not change the expression of GFP protein in non-intestinal cells, but only affected the expression in intestinal cells, suggesting a tissue-specific function for the GATA element (Supplementary Figure S3). These results indicate that the GATA element, which is likely to bind ELT-2 protein, is necessary in multiple genes for activation of transcription in intestinal cells in response to high zinc. Furthermore, the close proximity of the GATA element and the HZA element suggest these sequences may cooperate to mediate the cell type-specific response to high dietary zinc.

### A combination of the HZA and GATA elements was sufficient to mediate transcriptional activation in response to high zinc

The observation that the HZA element and the GATA element were necessary for transcriptional activation in response to high dietary zinc suggested that a combination of these elements might be sufficient to mediate this transcriptional response. To test this hypothesis, we used the *pes-10* promoter because it is established to be responsive to the addition of heterologous enhancers (47). A 62-bp fragment of the *mtl-1* promoter containing the HZA element and the GATA element was inserted upstream of the minimal *pes-10* promoter that drives the expression of GFP with a nuclear localization sequence. Transgenic animals containing the *pes-10* promoter with zero or one copies of this fragment did not display detectable GFP expression with or without supplemental zinc (data not shown). By contrast, transgenic animals containing the *pes-10* promoter with three copies of this fragment displayed low level GFP expression in the nucleus of intestinal cells in the absence of supplemental zinc; GFP protein expression was strongly induced specifically in intestinal cells in response to 100 μM supplemental zinc, and analysis of GFP mRNA showed an ∼18-fold increase in levels in response to high zinc (Figure 5B and C). Thus, the *pes-10* minimal promoter is not zinc inducible, whereas the addition of three copies of the HZA and GATA elements confers zinc inducibility in intestinal cells.

To characterize the metal specificity of this response, we analyzed these transgenic animals cultured with cadmium or copper. Supplementation with 50 μM cadmium caused induction of GFP protein expression in intestinal cells, and the analysis of GFP mRNA levels revealed an ∼2-fold increase (Figure 5D and E). By contrast, supplementation with copper did not induce GFP protein expression (data not shown). These results suggest that the *cis*-regulatory module composed of the HZA and GATA elements is sufficient to mediate transcriptional activation in intestinal cells in response to zinc and cadmium, but not to other metals, such as copper.

### Bioinformatic analysis of the HZA and GATA elements can be used to discover new zinc-regulated genes

The finding that four different genes regulated by high dietary zinc contained a HZA element and a nearby GATA element, and the observation that this combination of sequence elements was sufficient to mediate regulation by high zinc, led us to hypothesize that the presence of these *cis*-regulatory sequences could be used to predict additional zinc-responsive genes. To investigate this hypothesis, we established formal criteria for candidate sequence modules and used bioinformatic techniques to search the fully sequenced *C. elegans* genome for the genes that satisfy these criteria (Figure 6A). The criteria were as follows: (i) Genes contain sequence elements that have statistically significant similarity to the HZA element (Figure 1B) and the GATA element (38) positioned in the intergenic region upstream of the predicted translation start site. (ii) The HZA element and GATA element are separated by no more than 20 bp. (iii) These elements are conserved in the orthologous gene in other nematode species and these elements are part of *cis*-regulatory modules that are found at multiple locations within the genome (45). (iv) The length of promoter fragments was limited to 1.5 kb upstream of the translation start site. In *C. elegans*, 24 candidate genes met these criteria (Figure 6B). The list of genes includes known zinc and/or cadmium-responsive genes, such as *mtl-2* and *cdr-1*, which validates this approach. It is notable that the extent of overlap between the GATA and HZA elements varied from highly overlapping to non-overlapping, all four possible combinations of strand orientations were observed (−/−, −/+, +/− and +/+), and the distance to the start of translation varied from 59 to 1483 bp. Furthermore, the sequences of the HZA and GATA element displayed a range of similarities to the weight matrix. These properties might influence the sensitivity and magnitude of the response to excess zinc. However, the functional significance of different degrees of overlap, different orientations, different distances from the translation start site and different sequences are currently unknown.

The list of 24 genes did not include all known zinc inducible genes, such as *cdf-2*, indicating that the list is not comprehensive. This is presumably a result of the stringent criteria, especially that enhancers are part of *cis*-regulatory modules that are conserved during evolution. To generate a more comprehensive list, we conducted the analysis without considering evolutionary conservation or the *cis*-regulatory modules and identified a list of 116 genes that includes *mtl-1*,*mtl-2* and *cdf-2* (Supplementary Table S2).

To determine how reliably this approach identified genes activated by high dietary zinc, we analyzed five randomly selected genes that have not been characterized and one gene, *cdr-1*, that is reported to be cadmium inducible (48). Wild-type animals were cultured with 0 or 200 μM supplemental zinc, and mRNA levels of each gene were determined by qRT-PCR. *cdr-1* expression was induced ∼60-fold in response to high dietary zinc (Figure 6C), supporting the conclusion that the combination of the HZA and GATA element mediate induction in response to both dietary zinc and cadmium. Two newly identified genes, *F31E9.6* and *C33A11.4*, displayed induction that was not statistically significant. Three newly identified genes, *M02D8.6*,*K01A12.4* and *F13D11.4*, displayed statistically significant increases in mRNA levels of 3.3-, 3.4- and 6.6-fold, respectively, in response to high dietary zinc (Figure 6C). These results indicate that searching for HZA and GATA elements is an effective method to identify new genes that are regulated by high dietary zinc.

### The activities of *elt-2* and *mdt-15* were necessary for transcriptional activation by high zinc

**Figure 7.**
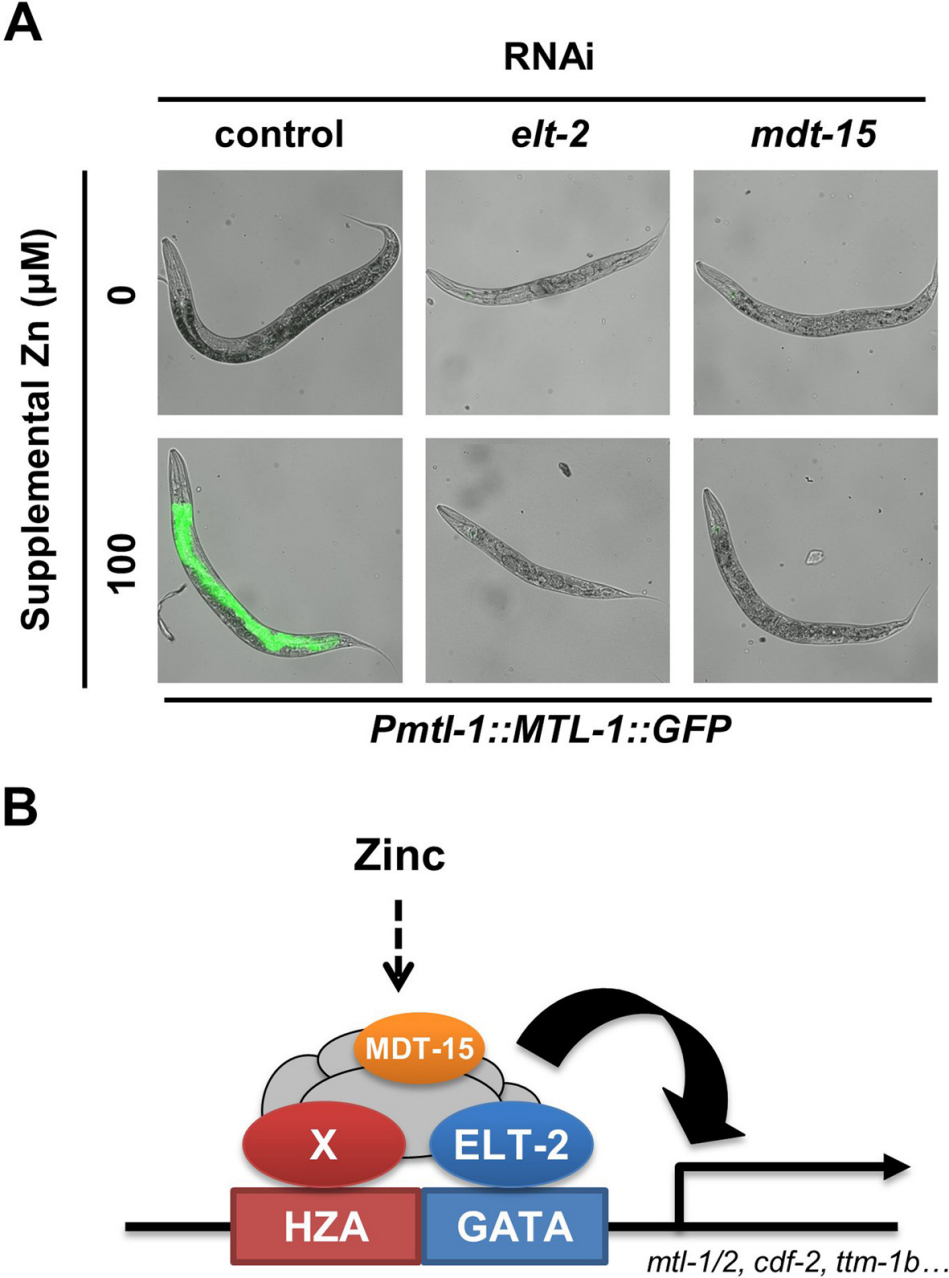
ELT-2 and MDT-15 were necessary for transcriptional activation in response to high zinc. (A) *Pmtl-1::MTL-1::GFP* transgenic animals were fed RNAi bacteria that targets the indicated genes starting from the L1 stage. Animals at the L4/young adult stage were cultured with 0 or 100 μM supplemental zinc and imaged by fluorescence microscopy. Bright field images are overlaid with GFP fluorescence signal (green) that were captured with the identical settings and exposure times. (B) A model of transcriptional activation by high levels of dietary zinc in intestinal cells. The black line indicates genomic DNA, boxes show the HZA and GATA elements, and the solid arrow indicates the transcription start site of zinc-responsive genes, such as *mtl-1, mtl-2, cdf-2* and *ttm-1b*. We propose that ELT-2 directly binds the GATA element and an unknown DNA-binding transcription factor directly binds the HZA element (X). MDT-15 is a component of an activation complex assembled at this location. Dietary zinc is taken up by intestinal cells and activates this complex (dotted arrow), and the activated complex causes transcriptional induction (curved arrow).

We hypothesized that the HZA and GATA elements mediate the effect of high dietary zinc by interacting directly with DNA-binding transcription factors, which in turn recruit coactivator proteins to induce transcription. To identify such factors, we used RNAi to reduce the activity of candidate genes and monitored expression of zinc-activated promoters. Because candidate genes may have multiple functions and be required for early development or survival, culturing with RNAi was initiated at the first larval (L1) stage. Because the GATA element is predicted to bind the ELT-2 transcription factor, and ELT-2 function is critical for expression of genes in intestinal cells (38), we analyzed *elt-2*. Transgenic animals cultured on bacteria that express *elt-2* RNAi generated progeny, indicating they are capable of substantial growth and metabolic activity, although they displayed reduced body size compared to animals cultured on control bacteria. When cultured with 100 μM supplemental zinc, animals fed control bacteria displayed strong GFP expression from the *mtl-1* promoter, whereas animals fed *elt-2* RNAi bacteria did not display GFP induction (Figure 7A). These results suggest that the ELT-2 transcription factor is necessary for activation of gene expression in response to high levels of zinc. An *elt-2* loss-of-function mutation was previously shown to block activation in response to cadmium (37). The mediator subunit MDT-15, which is predicted to be a component of a coactivator complex, has been demonstrated to play a role in induction of metal-responsive genes (36). To determine whether *mdt-15* is required for gene activation in response to high zinc in intestinal cells, we cultured transgenic animals with *mdt-15* RNAi bacteria. *mdt-15* RNAi disrupted induction of GFP expression from the *mtl-1* promoter in intestinal cells in the presence of 100 μM supplemental zinc (Figure 7A). To determine if *elt-2* and *mdt-15* are also necessary for induction of other zinc-responsive genes, we analyzed transgenic strains with the *cdf-2* and *ttm-1b* promoters driving expression of GFP (*Pcdf-2(WT)::GFP* and *Pttm-1b(-4kbWT)::GFP*). Compared to strains fed control bacteria, strains fed *elt-2* or *mdt-15* RNAi bacteria displayed reduced induction of GFP (data not shown). Together, these results suggest that both ELT-2 and MDT-15 are essential components of a transcriptional complex required for intestinal-specific activation in response to high dietary zinc.

## DISCUSSION

### Identification of an enhancer element that increases transcription in response to high dietary zinc and cadmium

We previously demonstrated that the zinc transporter genes *cdf-2* and *ttm-1b* display increased levels of transcripts in response to high dietary zinc (17,31). This regulation occurs at the level of transcription initiation, since the promoters of these genes can confer regulation on the heterologous GFP coding sequence. To characterize mechanisms that mediate this regulation, we used a combination of bioinformatic and functional approaches. In addition, we characterized the *mtl-1* and *mtl-2* genes, which are induced by a variety of stresses in *C. elegans*, including dietary zinc (35,36). Bioinformatic studies revealed that a similar 15 bp sequence, the HZA element, was present in the promoters of these four different genes. While protein and RNA coding sequences are typically highly conserved during evolution, non-coding sequences are typically much more divergent, unless they have a specific function. Sequence alignment analyses demonstrated that the HZA element of *mtl-1, mtl-2* and *cdf-2* is conserved between nematode species including *C. elegans*,*C. briggase*,*C. remanei* and *C. brenneri*. Thus, we hypothesized that this sequence might mediate regulation by zinc.

To directly evaluate the function of the HZA, we used site-directed mutagenesis to manipulate the sequence of a promoter and assayed for function *in vivo* using transgenic animals. We first defined promoter fragments of *cdf-2, ttm-1b* and *mtl-1* that contained the HZA element and were sufficient to mediate regulation by high zinc. When the HZA element was mutated, induction in response to high zinc was strongly reduced in all three genes. These results indicate that the HZA element is necessary for induction in response to high zinc. To rigorously test the model that the HZA mediates regulation by zinc, we determined if this element was sufficient to confer regulation on a minimal promoter. The insertion of three copies of the HZA region into the *pes-10* promoter resulted in a synthetic promoter that mediated transcriptional induction in response to high zinc specifically in intestinal cells. The findings that the HZA element is necessary in the context of native promoters to mediate HZA, and the HZA region is sufficient in a minimal promoter to mediate intestine-specific activation in response to high zinc, define the HZA region as an enhancer element. Enhancers are characterized by the ability to function at a variable distance from the transcription start site and in either orientation. Consistent with this model, the HZA element in native promoters functions at a range of distances from the start site (−194 bp in *cdf-2* to −2644 bp in *ttm-1b*) and in either orientation.

To characterize the metal specificity of the HZA element, we analyzed copper, a physiological metal in animals. The zinc-regulated genes *cdf-2, ttm-1b, mtl-1* and *mtl-2* were not induced by high dietary copper, indicating this response has some degree of metal specificity and these promoters are not simply responding to the stress of high metal toxicity. We also analyzed cadmium, a non-physiological metal in animals that is an environmental toxin; cadmium is structurally similar to zinc, since they are in the same column of the Periodic Table. Interestingly, all four genes were induced by dietary cadmium (41). Cui *et al.* have analyzed the transcriptional response to high dietary cadmium and identified a list of induced genes (41). By analyzing the promoters of these genes, we determined they contain versions of the HZA element, suggesting that the HZA element may mediate the response to high cadmium. Consistent with this model, we demonstrated that the HZA region was sufficient to confer intestine-specific cadmium responsiveness to the minimal *pes-10* promoter. These findings suggest that intestinal cells respond to high zinc and high cadmium by utilizing the same HZA enhancer element. These discoveries suggest a possible mechanism for cadmium toxicity. One consequence of cadmium toxicity is symptoms of zinc deficiency (49). Cadmium is known to displace zinc in zinc-dependent proteins, and this has been proposed to be a mechanism whereby cadmium causes symptoms of zinc deficiency. Our results suggest the possibility that cadmium activates the transcriptional response to excess zinc via the HZA element, which includes increased zinc excretion, chelation and storage. This transcriptional response may cause zinc deficiency. Both mechanisms could act in parallel, with cadmium displacing zinc in physiological binding sites and triggering a transcriptional response that reduces the pool of available zinc.

The HZA element from *C. elegans* has a substantially different sequence compared to previously characterized elements that mediate a response to zinc in other organisms. The analysis of *cdf-2, ttm-1b, mtl-1* and *mtl-2* identified a 15-bp weight matrix of the HZA element that includes 12 positions with a high frequency of a single nucleotide. By including 26 additional genes that were defined by cadmium regulation, we refined the weight matrix of the HZA element to 14 positions including 7 with a high frequency of a single nucleotide; the core sequence is 5′-CANAAAC-3′. This *C. elegans* sequence is quite different from the ZRE sequence that binds yeast ZAP1 (5′-ACCYYNAAGGT-3′) (19) and the MRE sequence that binds mammalian MTF-1 (5′-TGCRCNCGGCCC-3′) (23). These studies of *C. elegans* contribute to understanding both the similarities and diversity of the response to zinc in different organisms, since the mammalian MRE and the *C. elegans* HZA both regulate induction of metallothionein genes, although the enhancers have different sequences. Further studies are necessary to define the complete set of genes regulated by high zinc in *C. elegans*, which is important for determining the extent to which animals have evolved similar or different strategies to protect against excess zinc.

### A combination of GATA and HZA enhancer elements mediate intestine-specific transcriptional induction in response to high zinc

Intestinal cells are a major site of zinc metabolism in animals. In *C. elegans*, 20 intestinal cells mediate dietary uptake, storage, excretion and secretion of zinc into the body cavity to supply other cell types. Multiple zinc transporters and both metallothioneins are abundantly expressed in intestinal cells, and these genes are induced by zinc specifically in intestinal cells (17,33,37). For example, *ttm-1b* is expressed in multiple cell types including head neurons, seam cells, hypodermis, vulva and intestinal cells, but high dietary zinc induces *ttm-1b* transcripts only in intestinal cells (17). To characterize the mechanism of intestine-specific induction in response to high zinc, we searched for DNA sequences near the HZA and identified a GATA sequence element in *ttm-1b, mtl-1, mtl-2* and *cdf-2*. Mutagenesis analysis demonstrated that the GATA element was necessary for transcriptional activation of *mtl-1, cdf-2* and *ttm-1b* in response to high levels of zinc. Furthermore, the GATA element in combination with the HZA element was sufficient to mediate intestine-specific expression of the minimal *pes-10* promoter in response to high zinc. Similar to the HZA element, the GATA element was positioned at variable distances from the translation start site in different promoters and was found in both orientations, suggesting it functions as an enhancer element.

The ELT-2 transcription factor is a master regulator of intestinal cell identity; loss of *elt-2* function early in development causes a failure of intestinal cell differentiation, and essentially all of the genes expressed in intestinal cells are activated at the transcriptional level by binding ELT-2 protein (38). ELT-2 is a DNA binding protein that interacts with the DNA sequence GATA. Based on these results, we propose that the GATA element is an ELT-2 binding site that mediates intestine-specific expression, whereas the HZA element mediates activation in response to high dietary zinc (Figure 7). *mtl-1* and *mtl-2* are expressed in intestinal cells and have been reported to contain multiple predicted ELT-2 binding sites in their promoter regions; functional studies have indicated that only one or two of these sites are necessary for transcription (37). Interestingly, the GATA element that we identified in *mtl-1* because it is adjacent to the HZA is identical to the predicted ELT-2 binding site in *mtl-1* that was previously shown to be functionally significant. Recent studies in *C. elegans* have demonstrated that the transcriptional response to iron, a physiological metal, and heme, which is involved in iron metabolism, require predicted ELT-2 binding sites (50,51). In combination with our data, these findings suggest that intestinal-specific transcriptional activation in response to dietary metals is mediated by an intestine-specific enhancer element that binds ELT-2 and a nutrient-specific enhancer element, such as the HZA element for zinc or the iron-dependent element for iron.

### Identification of novel genes activated by high zinc

Based on the discovery that four genes that are induced by high zinc contain a combination of an HZA element and a GATA element in the promoter, we reasoned that searching the genome for this combination of enhancer elements could identify novel genes that are regulated by zinc. The *C. elegans* genome is completely sequenced and well annotated, so we focused our search on the predicted promoters of the ∼19 000 predicted *C. elegans* genes. We developed a weight matrix for the HZA element and the GATA element, and searched for promoters that contained both elements in close proximity. The search was designed to be stringent but not comprehensive, and it identified one of the four known genes, *mtl-2*, but not the other three known genes. In addition, we identified 23 genes that are candidates for zinc regulation. This list included several genes linked to metal homeostasis: *cdr-1* was identified based on induction by high cadmium, *Y54G9A.4* is predicted to encode a zinc transporter in the ZIP family and *ugt-54* is predicted to encode a UDP-glucuronosyl transferase that functions in the xenobiotic response. To determine if candidate genes are actually regulated by zinc, we analyzed five novel genes; these genes displayed from 2- to 7-fold induction in response to high zinc, and in three cases the induction was statistically significant. We conclude that this approach is effective in identifying novel genes that are induced by high zinc, establishing the foundation for future research that will determine if these genes play functional roles in the homeostatic response to zinc toxicity. Furthermore, these findings support the model that the combination of the tissue-specific GATA element and the nutrient-responsive HZA element mediate activation in response to high dietary zinc.

### ELT-2 and MDT-15 promote the transcriptional response to high zinc

We hypothesized that the zinc-responsive enhancer elements directly interact with DNA binding transcription factors, which assemble a transcriptional activation complex in response to high zinc. To investigate this hypothesis, we analyzed candidate genes. *elt-2* was analyzed because ELT-2 protein is predicted to bind the GATA element and is known to regulate expression of intestinal genes. Consistent with this model, reducing the activity of *elt-2* after the developmental stage when it is required to form the intestine abrogated the induction of zinc-responsive genes. This result is complementary with previous studies indicating that the ELT-2 transcription factor plays a role in gene expression in response to cadmium, iron and heme (37,50,51). Thus, ELT-2 may function in the homeostasis of many metals and metal-related factors. However, ELT-2 expression is not responsive to zinc levels, suggesting that another factor senses and responds to high zinc, while ELT-2 is likely to play a permissive role by mediating intestine-specific expression.

We propose that the HZA element directly interacts with a DNA-binding transcription factor, and the activity of this transcription factor responds to high zinc. To identify this transcription factor, we compared the sequence of the HZA element with a database of known transcription factor binding motifs; the HZA element was most similar to binding sites of the fork-head family of transcription factors (data not shown). DAF-16 was a candidate for interacting directly with the HZA, because it is a fork-head transcription factor that is abundantly expressed in intestinal cells and induced by a variety of stresses including metal exposure (52). However, the previously characterized DAF-16 binding sites in *mtl-1* (52) did not correspond with the HZA element, and reducing the activity of *daf-16* by RNAi did not affect *mtl-1* induction in response to high zinc (data not shown). These studies establish a foundation for future experiments to identify the transcription factor that binds the HZA element by a combination of DNA binding assays and functional tests for zinc-inducible gene expression.

We propose that the DNA-binding transcription factors assemble a complex of coactivators to promote gene expression. For example, mammalian MTF-1 functions in a complex with other transcription factors and cofactors (53). The mediator complex protein MDT-15 is involved in regulating the expression of detoxifying enzymes in response to ingested materials, including metals (36). Thus, we hypothesized that *mdt-15* might play a role in the response to high zinc. Consistent with this model, MDT-15 was necessary for zinc-responsive transcriptional activation, suggesting that the mediator complex is assembled at these promoters to activate transcription in response to high zinc. We propose that ELT-2 binding to the GATA element and an unknown transcription factor binding to the HZA element recruits the mediator complex to activate transcription in response to high dietary zinc (Figure 7B). These findings define the logic of combinatorial control by multiple enhancer elements that is responsible for tissue-specific and environmentally responsive gene expression.

## SUPPLEMENTARY DATA

Supplementary Data are available at NAR Online.

SUPPLEMENTARY DATA
