# Supplementary material for: A modular system of DNA enhancer elements mediates tissue-specific activation of transcription by high dietary zinc in C. elegans
Source: Nucleic Acids Res. 2014 Dec 30;43(2):803–16. doi: 10.1093/nar/gku1360 (PMC4333406; doi:10.1093/nar/gku1360)
Supplement: SUPPLEMENTARY DATA [file supp_43_2_803__index.html]

A modular system of DNA enhancer elements mediates tissue-specific activation of transcription by high dietary zinc in C. elegans — A modular system of DNA enhancer elements mediates tissue-specific activation of transcription by high dietary zinc in C. elegans — SUPPLEMENTARY DATA 

# A modular system of DNA enhancer elements mediates tissue-specific activation of transcription by high dietary zinc in *C. elegans*

## SUPPLEMENTARY DATA

**Files in this Data Supplement:**

- SUPPLEMENTARY DATA
