# Supplementary material for: A modular system of DNA enhancer elements mediates tissue-specific activation of transcription by high dietary zinc in C. elegans
Source: Nucleic Acids Res. 2014 Dec 30;43(2):803–16. doi: 10.1093/nar/gku1360 (PMC4333406; doi:10.1093/nar/gku1360)
Supplement: SUPPLEMENTARY DATA [file supp_gku1360_nar-02770-x-2014-File009.pdf]

**A**

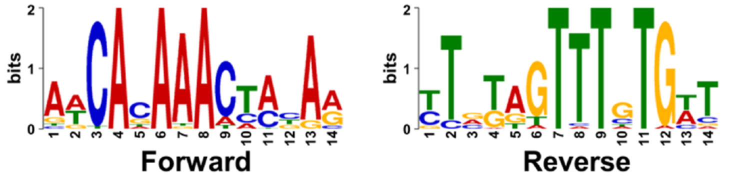


**B**

| Gene | | Position | Strand | Sequence | *p*-value |
|---|---|---|---|---|---|
| *C27H5.4* | | -1598 | - | AACACAAACTACAA | 7.36E-08 |
| *T08G5.1* | | -302 | - | ATCACAAACTAGAG | 7.36E-08 |
| *T08G5.10* | *mtl-2* | -292 | + | ATCACAAACTAGAG | 7.36E-08 |
| *T26H2.5* | | -143 | + | ATCACAAACTAGAA | 1.66E-07 |
| *K11G9.6* | *mtl-1* | -313 | + | ATCAGAAACTAGAG | 5.34E-07 |
| *F35E8.11* | *cdr-1* | -125 | + | AACAGAAACTACAA | 5.34E-07 |
| *Y39B6A.24* | | -1144 | - | AACAGAAACTACAA | 6.15E-07 |
| *F28D1.3* | *thn-1* | -1221 | - | AACACAAACTCCGA | 6.78E-07 |
| *T10B9.10* | *cyp-13A7* | -135 | + | AACACAAACCATAA | 7.66E-07 |
| *T10B9.1* | *cyp-13A4* | -131 | + | AACACAAACCATAA | 7.66E-07 |
| *R04D3.1* | *cyp-14A4* | -127 | + | AACACAAACTCTAC | 1.14E-06 |
| *Y46G5A.24* | *bcmo-1* | -294 | + | AACACAAACAACAA | 1.25E-06 |
| *T16G1.6* | | -86 | + | GACAGAAACCACAG | 1.37E-06 |
| *AC3.7* | *ugt-1* | -218 | + | GGCACAAACTCTAA | 2.18E-06 |
| *F08F8.5* | *numr-1* | -705 | + | TGCACAAACCACAG | 2.59E-06 |
| *T18D3.3* | *cdf-2* | -194 | - | ATCATAAACTAGAA | 3.90E-06 |
| *F56A4.5* | | -118 | - | CGCACAAACCATAG | 3.90E-06 |
| *C17H1.8* | | -548 | - | AGCACAAAACACAA | 4.25E-06 |
| *F41B5.2* | *cyp-33C7* | -143 | + | ATCATAAACTACGG | 5.80E-06 |
| *C02A12.1* | *gst-33* | -143 | - | GGCACAAACTAGGC | 6.20E-06 |
| *B0507.8* | | -306 | - | GACAGAAACTCCGC | 1.08E-05 |
| *F53C3.12* | *bcmo-2* | -862 | - | ATCAGAAAATCGAA | 1.15E-05 |
| *T10B9.2* | *cyp-13A5* | -153 | + | AACACAAATCATAG | 1.26E-05 |
| *F35E8.8* | *gst-38* | -202 | - | ATCAAAGACTAGAG | 2.57E-05 |
| *Y59E9AR.4* | *thn-5* | -373 | - | TTCAAAAACTATAG | 3.40E-05 |
| *C08E3.6* | *fbxa-163* | -64 | - | AACAGAAACAAAAA | 4.96E-05 |
| *F28D1.4* | *thn-3* | -61 | + | CTCATAAAATCCAG | 5.58E-05 |
| *C31B8.4* | | -746 | - | ATTACAAATTCCAA | 9.40E-05 |
| *C17H1.3* | | -1057 | + | AACAAAAAAACCAC | 1.03E-04 |

**Figure S1.** The HZA element is present in genes induced by high cadmium

(A) A 14 base pair sequence logo of the HZA element identified by MEME shown in the forward and reverse orientation. The promoter regions of the 29 cadmium-responsive genes [41] in panel B were analyzed. The height of the nucleotide at each position represents the frequency in the 29 DNA sequences. Panel A is identical to Figure 2C.

**(B)** Each gene has a genomic designation, and 19 genes also have a genetic name (right). The numbers indicate the position of the upstream base pair of the HZA element. Positions are relative to the predicted translation start site of the gene (ATG), where the A is defined as +1 and the preceding nucleotide is defined as -1. Strand (+ or -) indicate the same and opposite orientation relative to the direction of transcription, respectively. Sequence lists the 14 nucleotides that conform to the HZA element. P values indicate a statistical match score of the site computed from the comparison with the HZA shown in Panel A.

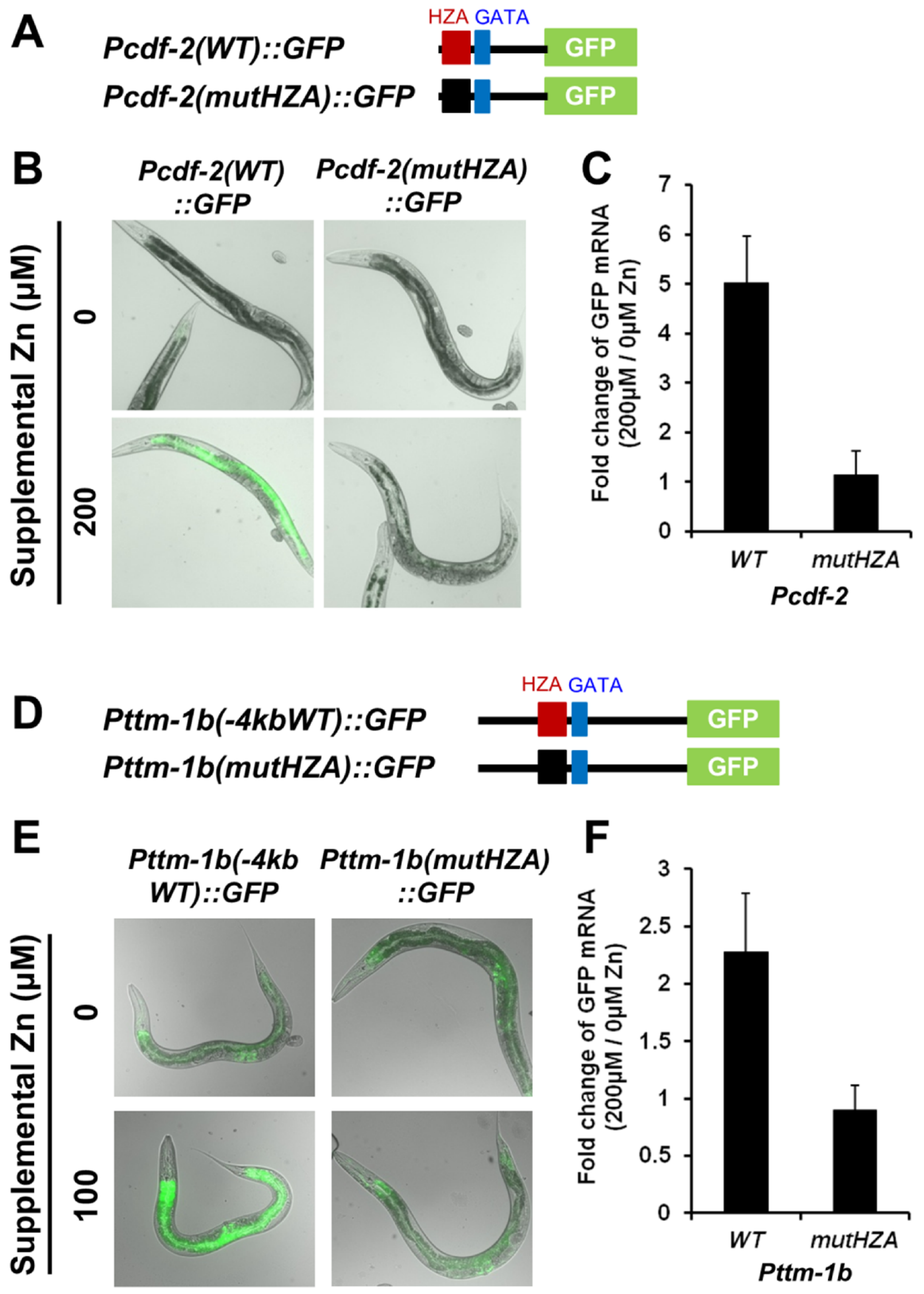


**Figure S2.** The HZA element was necessary for transcriptional induction of *cdf-2* and *ttm-1b* in response to high zinc (A, D) Diagrams (not to scale) of the *cdf-2* and *ttm-1b* promoter regions extending 204bp and 4067bp upstream of the translation start codons (black line), respectively, that were fused to the coding region for GFP. The fragments include the wild-type GATA binding site (blue box), and the wild-type HZA element (red box) or a mutated HZA element (black box) consisting of 15bp of scrambled nucleotide sequence. (B, E) Fluorescence microscope images of transgenic animals at L4/adult stages containing the indicated promoter constructs and cultured with 0μM, 100μM or 200μM supplemental zinc. Bright field images are overlaid with GFP fluorescence signal (green) that were captured with the identical settings and exposure times. Images in panel E (left) are identical to Figure 1B (middle). (C, F) Transgenic animals containing promoter fragments shown in panel A or D were cultured with 0μM or

200µM supplemental zinc, and GFP mRNA levels were analyzed by qRT-PCR. The bars indicate the fold change of GFP mRNA levels as the ratio between the level at 200µM and 0µM supplemental zinc. A positive value indicates induction in high zinc. Values are the average ± SEM of two independent experiments (panel C) or three independent experiments (panel F). GFP mRNA was consistently elevated at 200µM supplemental zinc in two or three independent trials, indicating this is a reproducible result. However, the combined data did not reach statistical significance at the level of $p<0.05$ because the values of the fold changes varied between the experiments.

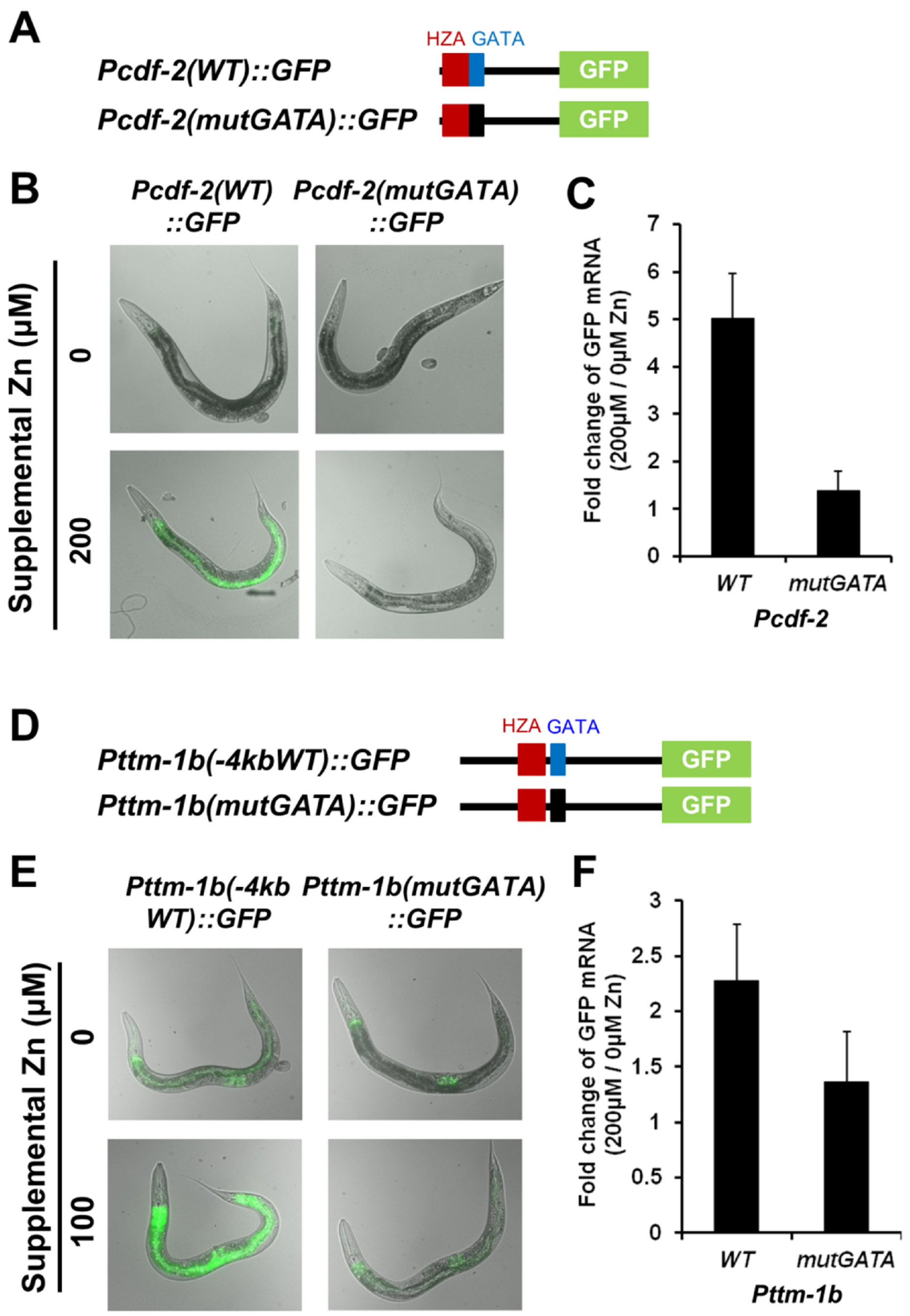


**Figure S3.** The GATA element was necessary for zinc-responsive transcriptional regulation of *cdf-2* and *ttm-1b*. (A, D) Diagrams (not to scale) of the *cdf-2* and *ttm-1b* promoter regions extending 204bp and 4067bp upstream of the translation start codons (black line), respectively, that were fused to the coding region for GFP. The fragment includes the wild-type HZA element (red box) and the wild-type GATA element (blue box) or the mutated GATA element (black box) consisting of scrambled nucleotide sequence. (B, E) Fluorescence microscope images of transgenic animals at L4/adult stages containing the indicated promoter constructs and cultured with 0µM, 100µM or 200µM supplemental zinc. Bright field images are overlaid with GFP fluorescence signal (green) that were captured with the identical settings and exposure times. Images in panel E (left) are identical to Figure 1B (middle). (C, F)

Transgenic animals containing promoter fragments shown in panel A or D were cultured with 0μM or 200μM supplemental zinc, and GFP mRNA levels were analyzed by qRT-PCR. The bars indicate the fold change of GFP mRNA levels as the ratio between the level at 200μM and 0μM supplemental zinc. A positive value indicates induction in high zinc. . Values are the average ± SEM of two independent experiments (panel C) or three independent experiments (panel F). GFP mRNA was consistently elevated at 200μM supplemental zinc in two or three independent trials, indicating this is a reproducible result. However, the combined data did not reach statistical significance at the level of $p<0.05$ because the values of the fold changes varied between the experiments.

## Table S1

| Strain[a] | Plasmid[b] | Genotype[c] |
|---|---|---|
| WU1418 | pHR21 | *amEx207(Pmtl-1(WT)::GFP;myo-3:mCherry)* |
| WU1420 | pHR21 | *amEx208(Pmtl-1(WT)::GFP;myo-3:mCherry)* |
| WU1421 | pHR21 | *amEx209(Pmtl-1(WT)::GFP;myo-3:mCherry)* |
| WU1394 | pKD8 | *amEx183(Pmtl-1(WT)::MTL-1::GFP::mtl-1 3'UTR;myo-3::mCherry)* |
| WU1422 | pHR22 | *amEx210(Pmtl-1(mutHZA)::GFP;myo-3:mCherry)* |
| WU1423 | pHR22 | *amEx211(Pmtl-1(mutHZA)::GFP;myo-3:mCherry)* |
| WU1424 | pHR22 | *amEx212(Pmtl-1(mutHZA)::GFP;myo-3:mCherry)* |
| WU1425 | pHR23 | *amEx213(Pmtl-1(mutGATA)::GFP;myo-3:mCherry)* |
| WU1426 | pHR23 | *amEx214(Pmtl-1(mutGATA)::GFP;myo-3:mCherry)* |
| WU1427 | pHR23 | *amEx215(Pmtl-1(mutGATA)::GFP;myo-3:mCherry)* |
| WU1395 | pID1 | *amEx184(Pmtl-1(mut(1-21))::MTL-1::GFP::mtl-1 3'UTR;myo-3::mCherry)* |
| WU1396 | pID1 | *amEx185(Pmtl-1(mut(1-21))::MTL-1::GFP::mtl-1 3'UTR;myo-3::mCherry)* |
| WU1428 | pHR24 | *amEx216(Pcdf-2(WT)::GFP;myo-3::mCherry)* |
| WU1429 | pHR25 | *amEx217(Pcdf-2(mutHZA)::GFP;myo-3::mCherry)* |
| WU1430 | pHR25 | *amEx218(Pcdf-2(mutHZA)::GFP;myo-3::mCherry)* |
| WU1431 | pHR25 | *amEx219(Pcdf-2(mutHZA)::GFP;myo-3::mCherry)* |
| WU1432 | pHR26 | *amEx220(Pcdf-2(mutGATA)::GFP;myo-3::mCherry)* |
| WU1433 | pHR26 | *amEx221(Pcdf-2(mutGATA)::GFP;myo-3::mCherry)* |
| WU1344 | pHR6 | *amEx154(Pttm-1b(-6kb)::GFP;myo-3::mCherry)* |
| WU1345 | pHR6 | *amEx155(Pttm-1b(-6kb)::GFP;myo-3::mCherry)* |
| WU1346 | pHR6 | *amEx156(Pttm-1b(-6kb)::GFP;myo-3::mCherry)* |
| WU1347 | pHR6 | *amEx157(Pttm-1b(-6kb)::GFP;myo-3::mCherry)* |
| WU1343 | pHR18 | *amEx153(Pttm-1b(-2.4kb)::GFP;myo-3::mCherry)* |
| WU1409 | pHR13 | *amEx198(Pttm-1b(-4kb)::GFP;myo-3::mCherry)* |
| WU1410 | pHR13 | *amEx199(Pttm-1b(-4kb)::GFP;myo-3::mCherry)* |
| WU1411 | pHR13 | *amEx200(Pttm-1b(-4kb)::GFP;myo-3::mCherry)* |
| WU1412 | pHR14 | *amEx201(Pttm-1b(-4kb)(mutHZA)::GFP;myo-3::mCherry)* |
| WU1413 | pHR14 | *amEx202(Pttm-1b(-4kb)(mutHZA)::GFP;myo-3::mCherry)* |
| WU1414 | pHR14 | *amEx203(Pttm-1b(-4kb)(mutHZA)::GFP;myo-3::mCherry)* |
| WU1415 | pHR20 | *amEx204(Pttm-1b(-4kb)(mutGATA)::GFP;myo-3::mCherry)* |
| WU1416 | pHR20 | *amEx205(Pttm-1b(-4kb)(mutGATA)::GFP;myo-3::mCherry)* |
| WU1417 | pHR20 | *amEx206(Pttm-1b(-4kb)(mutGATA)::GFP;myo-3::mCherry)* |
| WU1397 | pID24 | *amEx186(3XGATA&HZA::pes-10::NLS-GFP;myo-3::mCherry)* |
| WU1398 | pID24 | *amEx187(3XGATA&HZA::pes-10::NLS-GFP;myo-3::mCherry)* |

[a] The name of the strain containing the extrachromosomal array formed by injection of the indicated plasmid and a plasmid with a transformation marker. The wild-type N2 strain was injected in all cases.

[b] The name of the plasmid that contains the zinc-responsive promoter fragment.

[c] The name of the extrachromosomal array that contains the indicated plasmid and plasmid pCJF104 [*Pmyo-3::mCherry*], a coinjection marker that expresses mCherry from the muscle-specific *myo-3* promoter.

**Table S2. Candidate genes predicted by a bioinformatic search (without *C. elegans* Regulatory Module Detector (CERMOD) search criteria)**

| *Gene*[a] | | GATA Position[b] | HZA Position[c] |
|---|---|---|---|
| *B0412.2* | *daf-7* | -804 | -822 |
| *B0563.7* | | -1462 | -1448 |
| *C01C10.2a* | | -664 | -672 |
| *C03B1.10* | | -72 | -67 |
| *C03F11.1* | | -735 | -718 |
| *C06A5.1* | | -253 | -261 |
| *C06A5.9* | *rnf-1* | -348 | -343 |
| *C06C6.4* | *nhr-63* | -1071 | -1066 |
| *C06E2.5* | | -223 | -231 |
| *C07A9.11* | *ncx-7* | -133 | -146 |
| *C07G3.8a* | | -1243 | -1225 |
| *C07G3.8b* | | -401 | -383 |
| *C08E3.1* | | -1380 | -1375 |
| *C08E3.13* | | -128 | -136 |
| *C11D2.2* | | -210 | -224 |
| *C15A7.4* | | -202 | -200 |
| *C17B7.11* | *fbxa-65* | -143 | -151 |
| *C17F4.12* | | -39 | -22 |
| *C24A3.1* | | -70 | -89 |
| *C24A3.4* | | -387 | -382 |
| *C33A11.4b* | | -1034 | -1053 |
| *C34B4.1b* | *max-1* | -1377 | -1385 |
| *C35C5.5* | *lev-8* | -397 | -384 |
| *C42D8.5a* | *acn-1* | -393 | -388 |
| *C47F8.2* | *nhr-165* | -189 | -187 |
| *C47F8.8* | *nhr-81* | -1415 | -1420 |
| *C50B6.12* | *str-37* | -1454 | -1459 |
| *C50B6.7* | *amylase* | -1352 | -1350 |
| *D2023.1g* | | -155 | -146 |
| *D2023.3a* | | -1320 | -1315 |
| *F02E8.5* | *atg-16.1* | -1101 | -1099 |
| *F08D12.4* | | -352 | -367 |
| *F08F1.6* | *spp-13* | -284 | -279 |
| *F10A3.1* | | -151 | -146 |
| *F13D11.4* | | -184 | -173 |
| *F13H8.6* | | -1264 | -1272 |
| *F16A11.3a* | *ppfr-1* | -400 | -408 |
| *F17E9.11* | *lys-10* | -193 | -188 |
| *F18E9.1* | | -1205 | -1186 |
| *F20C5.5* | | -424 | -443 |
| *F22B3.5a* | | -897 | -905 |
| *F23D12.5* | | -687 | -682 |
| *F23H12.8* | *fipr-1* | -552 | -571 |
| *F26F2.9* | | -755 | -738 |

| | | | |
|---|---|---|---|
| *F28G4.1* | *cyp-37B1* | -102 | -97 |
| *F31E9.6* | | -791 | -809 |
| *F35E8.10* | | -936 | -923 |
| *F35E8.11* | *cdr-1* | -109 | -125 |
| *F38C2.4a* | | -845 | -840 |
| *F43B10.1* | | -1223 | -1214 |
| *F45C12.15* | | -410 | -418 |
| *F45D11.1* | | -852 | -847 |
| *F45D11.14* | | -308 | -303 |
| *F45D11.15* | | -329 | -324 |
| *F45D11.16* | | -329 | -324 |
| *F46F3.4c* | | -353 | -365 |
| *F46F3.4d* | | -829 | -841 |
| *F47B8.10* | | -1277 | -1262 |
| *F49E12.2* | *dod-23* | -913 | -931 |
| *F54D8.3b* | *alh-1* | -632 | -627 |
| *F55A4.8a* | | -500 | -514 |
| *F55B11.1* | | -1203 | -1201 |
| *F59B1.2* | | -1017 | -1033 |
| *F59B1.6* | | -650 | -637 |
| *F59B2.12* | | -935 | -943 |
| *H12D21.10a* | | -1022 | -1037 |
| *H12D21.6* | | -1046 | -1034 |
| *H27A22.1a* | | -225 | -220 |
| *K01A12.4* | | -107 | -102 |
| *K02A4.1* | *bcat-1* | -1467 | -1481 |
| *K02E7.6* | | -317 | -312 |
| *K04H4.5* | | -914 | -909 |
| *K11G9.6* | *mtl-1* | -323 | -313 |
| *M02D8.6* | | -105 | -100 |
| *M04C9.2* | | -59 | -67 |
| *M05D6.4* | *lact-4* | -220 | -212 |
| *M110.4e* | *ifg-1* | -186 | -194 |
| *R08A2.2* | | -543 | -531 |
| *R08A2.4* | *fbxa-147* | -385 | -400 |
| *R10H1.2a* | *srab-14* | -1475 | -1483 |
| *R12C12.8a* | | -1401 | -1420 |
| *T05B11.3* | *clic-1* | -606 | -592 |
| *T05B11.6* | *srr-10* | -141 | -158 |
| *T05G11.2* | *srbc-77* | -232 | -227 |
| *T08G5.1* | | -297 | -305 |
| *T08G5.10* | *mtl-2* | -297 | -292 |
| *T18D3.3* | *cdf-2* | -182 | -194 |
| *T18D3.4* | *myo-2* | -1203 | -1194 |
| *T21C9.7* | *srg-32* | -217 | -225 |
| *T21E8.1a* | *pgp-6* | -1410 | -1418 |
| *T21E8.1c* | | -239 | -247 |
| *T23B3.4* | *ckr-1* | -1067 | -1075 |

| | | | |
|---|---|---|---|
| *T25B9.7* | *ugt-54* | -649 | -663 |
| *T26H2.5* | | -156 | -145 |
| *W01A8.1a* | *mdt-28* | -34 | -47 |
| *W01A8.2* | | -859 | -857 |
| *W06G6.11b* | | -395 | -384 |
| *Y105C5A.13b* | | -130 | -138 |
| *Y105C5B.2* | *gcy-25* | -410 | -426 |
| *Y105E8A.24a* | | -1320 | -1315 |
| *Y22F5A.3b* | *ric-4* | -485 | -480 |
| *Y37D8A.3* | | -1167 | -1148 |
| *Y39A1B.2a* | | -631 | -644 |
| *Y43E12A.2* | | -1450 | -1433 |
| *Y54G2A.16* | | -357 | -339 |
| *Y54G9A.4* | | -73 | -81 |
| *Y64G10A.6* | | -616 | -611 |
| *Y71F9B.1* | | -969 | -964 |
| *ZC142.1* | *srt-13* | -983 | -978 |
| *ZC443.6* | *ugt-16* | -86 | -100 |
| *ZK265.8a* | | -1042 | -1040 |
| *ZK287.2a* | | -510 | -502 |
| *ZK287.2b* | | -1000 | -992 |
| *ZK688.6a* | *pcp-5* | -37 | -45 |
| *ZK813.3* | | -1017 | -1030 |
| *ZK909.4* | *ces-2* | -865 | -882 |

[a] Each gene has a genomic designation, and 44 genes also have a genetic name (right).

[b, c] Numbers indicate the position of the upstream base pair of the GATA element or the HZA element. Positions are relative to the predicted translation start site of the gene (ATG), where the A is defined as +1 and the preceding nucleotide is defined as -1. To search for the GATA element, we used a 12 bp weight matrix, and to search for the HZA element, we used a 15 bp weight matrix.
